# Supplementary figures and images for: The GravyTrain toolbox for molecular cell biology
Source: PLoS One. 2025 Sep 17;20(9):e0332438. doi: 10.1371/journal.pone.0332438 (PMC12443241; doi:10.1371/journal.pone.0332438)

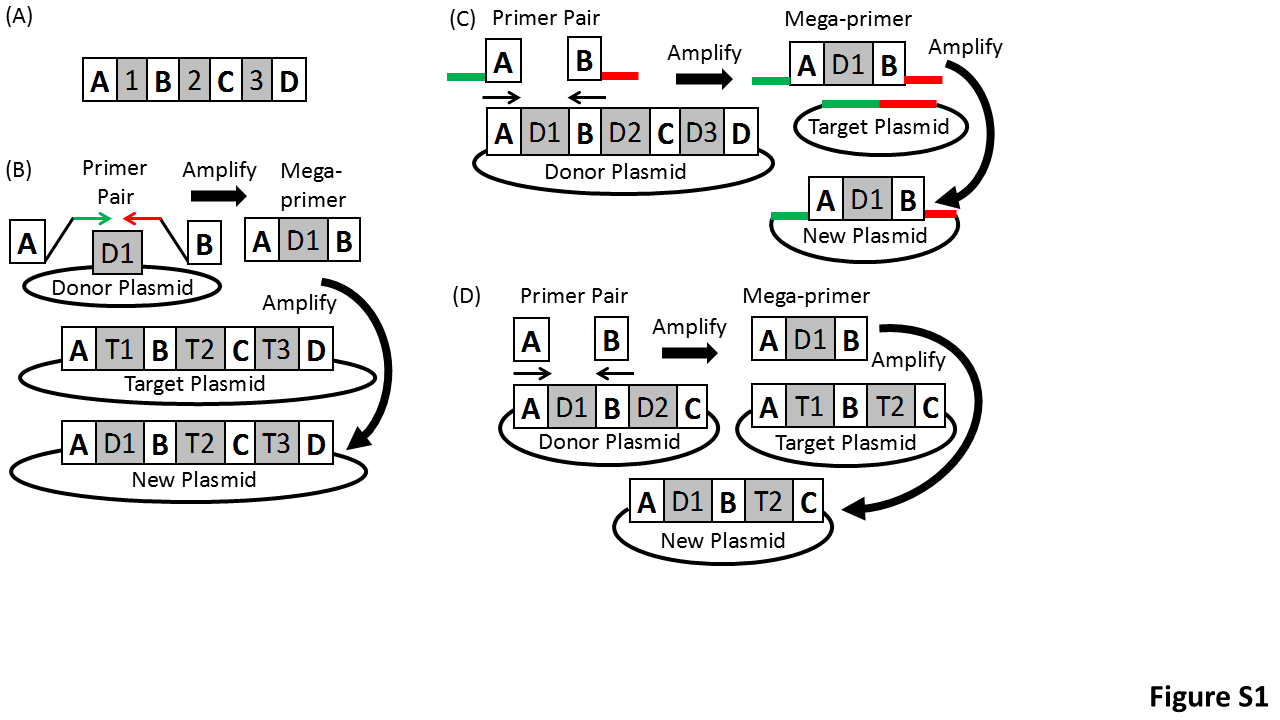

Supplement: S1 Fig — (A) Common layout of GravyTrain constructs. Gray background – GravyTrain genetic elements. White background – a pair of GravyTrain restriction-free hotspots flank each genetic element. (B) Import of an element from an external construct into a GravyTrain construct. First, a forward primer consisting of hotspot A sequence followed by sense homology to the 5’ end (green arrow) of the donated element (“D1”), and a reverse primer consisting of hotspot B reverse sequence followed by antisense homology to the 3’ end (red arrow) of D1, are used to amplify the element from a non-GravyTrain donor plasmid into a mega-primer. Note that primer design is tailored for the specific element but is independent of target plasmid. Then, the mega-primer is used to amplify the target GravyTrain plasmid containing an old element 1 (“T1”). This generates a new GravyTrain plasmid, wherein T1 is substituted with the imported D1. (C) Export of a GravyTrain to an external construct. First, a forward primer consisting of sense homology to the 5’ half of the target insertion site (green region) followed by Hotspot A sequence, and a reverse primer consisting of antisense homology to the 3’ half of the target insertion site (red region) followed by Hotspot B reverse sequence, are used to amplify the donated element 1 (“D1”) from a GravyTrain donor plasmid into a mega-primer. Note that primer design is tailored for target plasmid but is independent of the specific element. Then, the mega-primer is used to amplify the target non-GravyTrain plasmid, thereby generating a new non-GravyTrain plasmid containing exported element. (D) Shuffling of a GravyTrain element between GravyTrain constructs. First, a forward primer consisting of Hotspot A sequence, and a reverse primer consisting of Hotspot B reverse sequence, are used to amplify the element D1 from a donor GravyTrain plasmid into a mega-primer. Note that primer design is independent of shuffled element and both donor and target plasmids. Then, the [file pone.0332438.s004.tif]

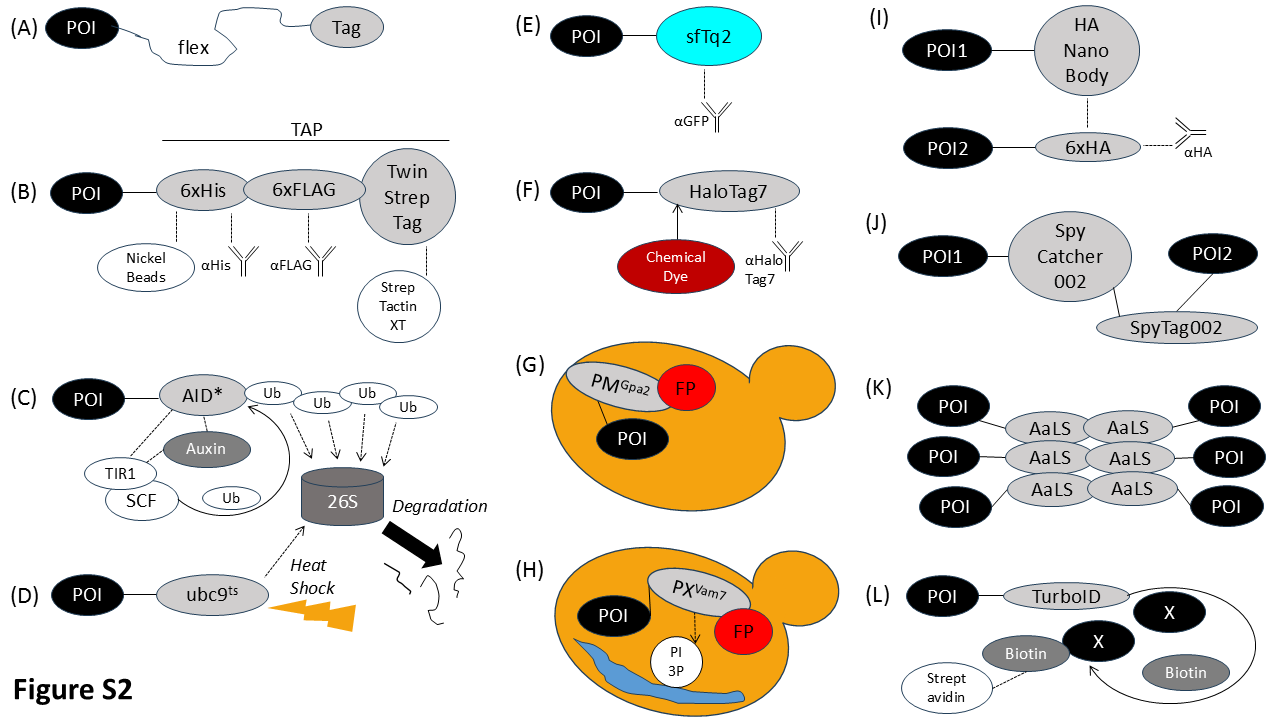

Supplement: S2 Fig — Representative examples of protein fusion tags for characterization or manipulation of a Protein Of Interest (POI). Antibodies and other interactors for post-lysis characterization are indicated next to tags. (A) a flex for separating POI from another fusion tag; (B) TAP – including 6xHis, 6xFLAG and TwinStrepTag – for affinity-based biochemical purification and identification; (C) AID* for auxin-induced, transient and quantitative SCFTIR1-mediated poly-ubiquitination and consequent degradation by the 26S proteasome; (D) ubc9ts for temperature-inflicted, complete degradation by the 26S proteasome; (E) superfolder mTurquoise2 (sfTq2) for constitutive cyan visualization; (F) HaloTag7 for transient, Halo chemical ligand-mediated visualization; (G) PMGpa2 for plasma membrane anchoring or- (H) PXVam7 for tethering to PI(3)P are fused to a fluorescent protein (“FP”) for in situ visualization; (I) HA nanobody for tethering of POI1 to another, HA-tagged POI2; (J) SpyCatcher002 for covalent binding of POI1 to another, SpyTag002-fused POI2; (K) AaLS for spatial condensation on the surface of a multimeric nanoparticle. (L) TurboID for biotin proximity labeling. (TIF) [file pone.0332438.s005.tif]

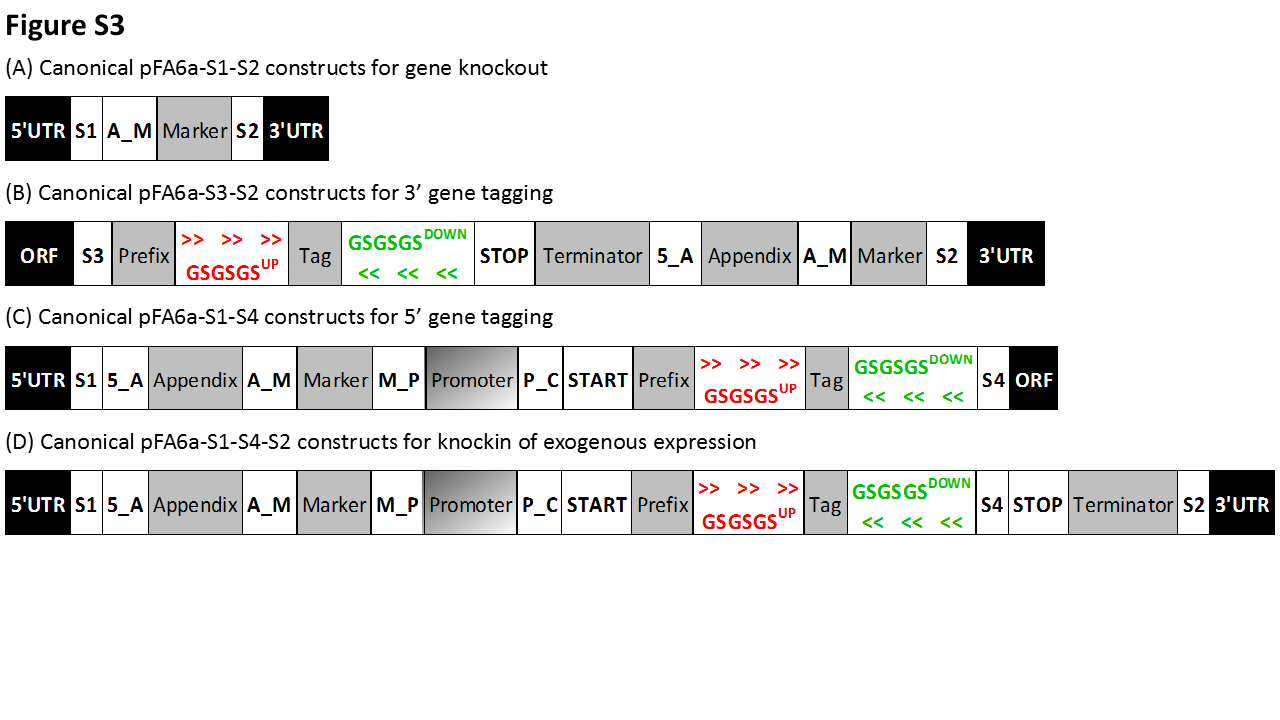

Supplement: S3 Fig — Common layouts, in 5’ to 3’ sense orientation, of specific types of canonical GravyTrain constructs for genome editing in yeast, following homologous recombination-mediated integration in a genomic locus. Black background – genomic elements. White background – GravyTrain restriction-free hotspots. Gray background – GravyTrain genetic elements. Marker – selection marker for yeast transformation. Appendix – additional genetic element for integration, to be used in conjunction with construct (not in use in constructs provided herein). Prefix – polypeptide part, such as a linker or degron, to be expressed in fusion with the main functional tag and fused endogenous ORF. Promoter and Terminator – drive expression of the ORF. GSGSGS_UP and GSGSGS_DOWN – short neutral linkers flanking main functional tag. START and STOP – start and stop codons, respectively, for ORF translation. (A) Canonical pFA6a-S1-S2 constructs for gene knockout. Amplified by forward sense S1 and reverse antisense S2 primers and integrated between endogenous 5’UTR and 3’UTR instead of endogenous ORF, thereby knocking out its expression. (B) Canonical pFA6a-S3-S2 constructs for 3’ gene tagging. Amplified by forward sense S3 and reverse antisense S2 primers and integrated between endogenous ORF and 3’UTR, thereby tagging the ORF at its C-terminus with the Prefix-Tag moiety and substituting endogenous terminator function with GravyTrain terminator within construct. (C) Canonical pFA6a-S1-S4 constructs for 5’ gene tagging. Amplified by forward sense S1 and reverse antisense S4 primers and integrated between endogenous 5’UTR and ORF, thereby tagging the ORF at its N-terminus with the Prefix-Tag moiety and substituting endogenous promoter function with GravyTrain promoter within construct. (D) Canonical pFA6a-S1-S4-S2 constructs for knock-in of exogenous expression. Amplified by forward sense S1 and reverse antisense S2 primers and integrated between endogenous 5’UTR and 3’UTR instead of endogenous ORF, there [file pone.0332438.s006.tif]

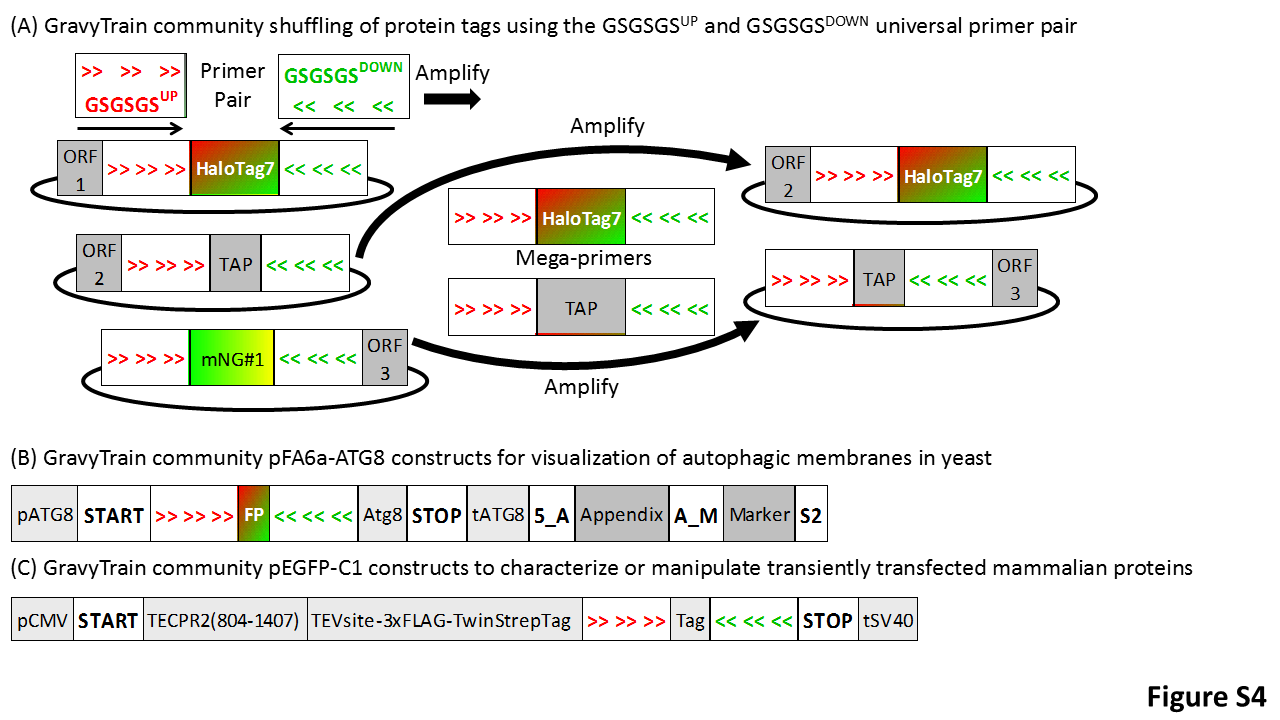

Supplement: S4 Fig — (A) Shuffling of GravyTrain protein fusion tags between different construct types: the universal GSGSGS_UP (red forward arrows) and GSGSGS_DOWN (green backward arrows) primer pairs are used to amplify mega-primers of GravyTrain tags (middle) from donor constructs of one type (left), and target them by another amplification to constructs from another type (pointed by diagonal arrows). This generates new constructs where the target tag is substituted by the tag from the donor tag. This shuffling capacity defines a community of GravyTrain constructs of different types – two examples of which follow below – beyond the sets of canonical yeast genetics constructs described above. (B) GravyTrain community pFA6a-ATG8 constructs allow visualization of autophagic membranes in yeast by expressing an extra copy of Atg8, fused N-terminally to a GravyTrain fluorescent protein (“FP”) flanked by GSGSGS_UP and GSGSGS_DOWN. Construct is amplified by forward sense primer annealing at the beginning of the native ATG8 promoter (“pATG8”) and a reverse antisense S2 primer, and genomically integrated between endogenous 5’UTR and 3’UTR instead of an endogenous ORF, thereby knocking out its expression. Included GravyTrain elements are described above. FP-Atg8 is expressed under control of native ATG8 promoter (“pATG8”) and terminator (“tATG8”). (C) GravyTrain community pEGFP-C1 constructs allow characterization or manipulation of a transiently transfected overexpressed mammalian protein like a fragment of TECPR2 (“TECPR2(804-1407)”), fused C-terminally to a TEV cleavage site followed by 3xFLAG and TwinStrepTag affinity tags, and a variable GravyTrain protein fusion tag (“Tag”) flanked by GSGSGS_UP and GSGSGS_DOWN. Upon transient transfection to a cell culture, protein and fused moieties are expressed as a single polypeptide under control of the CMV promoter (“pCMV”) and SV40 terminator (“tSV40”). (TIF) [file pone.0332438.s007.tif]

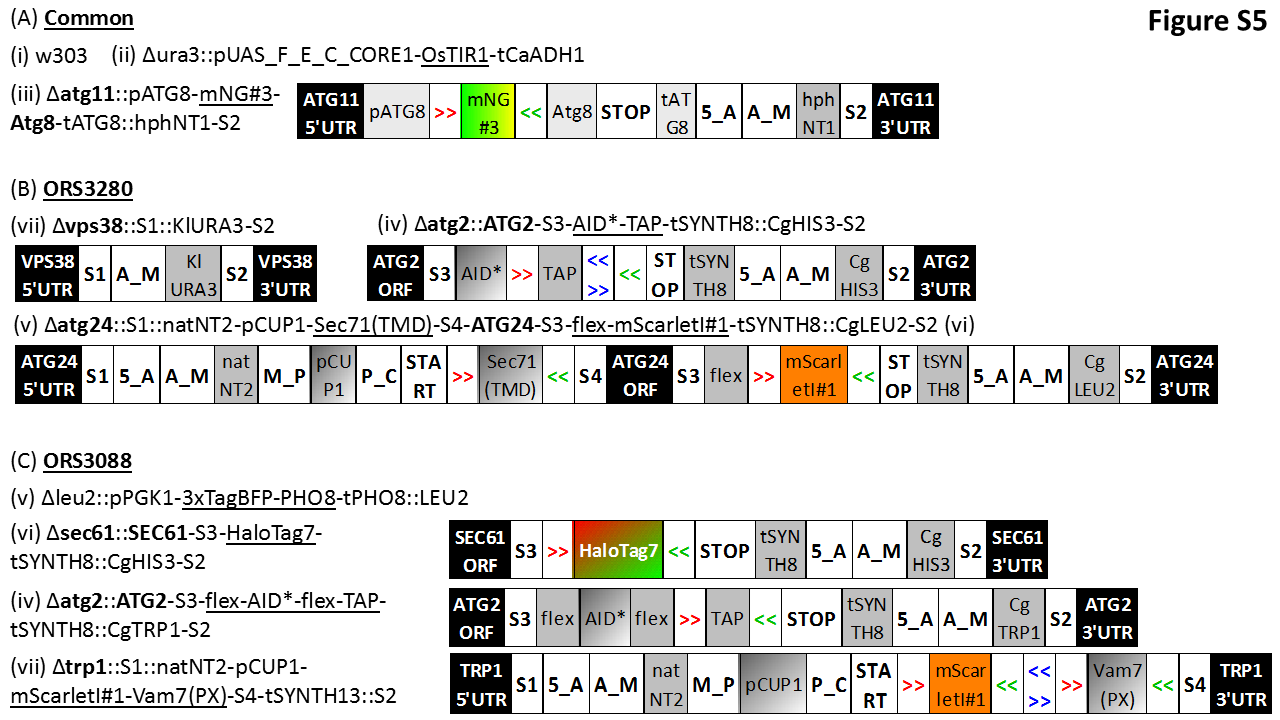

Supplement: S5 Fig — Presented are two genotypes of two strains – ORS3280 (B) and ORS3088 (C) – as examples for instrumentation of GravyTrain constructs in construction of a strain library for studying autophagy in yeast. (i-iv) are common to both strains (A): (i) w303 background for visualization of large nonselective autophagic membranes (not depicted); (ii) overexpression of non-GravyTrain TIR1 for AID*-mediate protein depletion (not depicted); (iii) integration of GravyTrain community ATG8 construct mNeonGreen-Atg8 (pOS222) for visualization of autophagic membranes, instead of the ORF of endogenous ATG11 to eliminate Atg11-mediated selective autophagy; (iv) C-terminal tagging of endogenous ATG2 ORF with GravyTrain construct of AID* (pOS343 for ORS3280, pOS525 for ORS3088) for transient partial auxin-mediated depletion of Atg2. (B) Strain ORS3280 was instrumented to establish that tethering the PI(3)P-binding protein Atg24 to the cytoplasmic ER membrane leaflet excessively opens the rim of the nonselective autophagic isolation membrane upon partial loss of Atg2-Atg18 complex activity, in a manner independent of endosomal PI(3)P: (v) C-terminal tagging of endogenous ATG24 ORF with GravyTrain construct of mScarletI (pOS249) for visualization of Atg24; (vi) N-terminal tagging of endogenous ATG24 ORF with GravyTrain construct of TMDSec71 (pOS553) for tethering of Atg24 to the cytoplasm leaflet of the ER membrane; (vii) knockout of VPS38 ORF for elimination of endosomal PI(3)P. (C) Strain ORS3088 was instrumented to establish the bona fide autophagic identity of aberrantly cup-shaped nonselective autophagic structures that form upon partial loss of Atg2-Atg18 complex activity, as Atg8, PI(3)P-positive projections from the vacuolar membrane towards the ER: (v) overexpression of non-GravyTrain TagBFP-Pho8, integrated at the LEU2 locus, to visualize the vacuolar membrane (not depicted); (vi) C-terminal fusion of endogenous ER protein Sec61 with GravyTrain construct of HaloTag7 (pOS442) for f [file pone.0332438.s008.tif]
