# Supplementary material for: The GravyTrain toolbox for molecular cell biology
Source: PLoS One. 2025 Sep 17;20(9):e0332438. doi: 10.1371/journal.pone.0332438 (PMC12443241; doi:10.1371/journal.pone.0332438)
Supplement: Attachment — Submitted filename: Response_to_Reviewers_auresp_2.pdf [file pone.0332438.s012.pdf]

Journal Requirements:

When submitting your revision, we need you to address these additional requirements.

1. Please ensure that your manuscript meets PLOS ONE's style requirements, including those for file naming. The PLOS ONE style templates can be found at https://journals.plos.org/plosone/s/file?id=wjVg/PLOSOne_formatting_sample_main_body.pdf and https://journals.plos.org/plosone/s/file?id=ba62/PLOSOne_formatting_sample_title_authors_affiliations.pdf

We made sure our manuscript meets PLOS ONE's style requirements.

2. We note that the grant information you provided in the 'Funding Information' and 'Financial Disclosure' sections do not match.

When you resubmit, please ensure that you provide the correct grant numbers for the awards you received for your study in the 'Funding Information' section.

Correct grant numbers are now provided to match 'Funding Information' and 'Financial Disclosure'.

3. Thank you for stating the following financial disclosure: "The authors have declared that no competing interests exist."

Please state what role the funders took in the study. If the funders had no role, please state: "The funders had no role in study design, data collection and analysis, decision to publish, or preparation of the manuscript."

If this statement is not correct you must amend it as needed.

Please include this amended Role of Funder statement in your cover letter; we will change the online submission form on your behalf.

The financial disclosure text was amended as follows:

"The authors have declared that no competing interests exist. The funders had no role in study design, data collection and analysis, decision to publish, or preparation of the manuscript."

4. We are unable to open your Supporting Information file [S1_File.zip]. Please kindly revise as necessary and re-upload.

The supporting ZIP file opens up correctly in our computers, both as originally submitted as well as when accessed from the link in the PDF produced by the editorial system upon submission. We may therefore have to coordinate the transfer of this file with the journal staff in person. Otherwise, we hope that the following shared link allows simple and easy access to the file:

https://drive.google.com/file/d/1QsjFuVJig0dHV2lAA17pQvPbw952h1jx/view?usp=sharing

Reviewer #1:

the abstract needs a little simplification as it is dense with more details and thus making it less overview in understanding to the scholarly community.

The simplification in this matter will be a better option so that it could be cool.

We agree and have now simplified the abstract.

The figures 1-5 needs clear annotation and it could be done to make it clear.

Thank you for this comment. Most of the graphical elements have been relocated to the supplementary information for the more advanced interested readership that would like to deepen their acquaintance with the toolbox. The main figure is now annotated for the broader readership.

A flowchart summarizing the workflow for GravyTrain construct generation and strain library development would enhance understanding.

Figure 1A, S1 schematically describe – together with the matching main text – the main actions taken for creating GravyTrain constructs, namely shuffling elements between donor and target GravyTrain constructs (Fig 1A, S1D), importing new elements from donor non-GravyTrain plasmids into GravyTrain target plasmids (Fig S1B) and exporting elements from donor GravyTrain into target non-GravyTrain plasmids (Fig S1C).

Figure S5 schematically describes – together with the matching main text – the steps taken for constructing representative yeast strains based on GravyTrain constructs for studying autophagy. The complete strain library was constructed in a similar fashion by modifying different gene combinations with various GravyTrain constructs as required by individual experiments in our study (PMID: 38447569).

Reviewer #2:

Dear Editors,

I have reviewed the manuscript titled "The GravyTrain Toolbox for Molecular Cell Biology" (Manuscript PONE-D-24-45023). The manuscript presents a potentially transformative toolbox for genetic manipulation in yeast and other model organisms. However, I identified several areas that require significant revision to enhance the manuscript's clarity, validation, and generalizability.

We thank the reviewer for appreciating the potential of our toolbox.

Key areas of concern include:

The abstract is overly technical and does not clearly frame the problem GravyTrain addresses.

We have simplified the abstract to concentrate on the key contributions of GravyTrain rather than the problems solved thereby.

The introduction lacks a strong comparison with alternative tools like CRISPR/Cas9 and SWAP-tag systems.

We do not portray the motivation to use GravyTrain as an alternative to CRISPR/Cas9 or SWAP-tag but rather as a complementary approach that directly continues the commonly employed pYM plasmid series legacy. As such, we maintain that the brief comparison included in the introduction is sufficient to put our toolbox in context, while a more elaborate introduction to alternatives is more suitable in scope of a literature review of different approaches for cloning and genetics. Accordingly, the reference to alternatives is also kept brief in the discussion.

The manuscript lacks sufficient experimental evidence to substantiate the claimed advantages of GravyTrain, such as reduced recombination errors and broad applicability. Robust comparative data are necessary.

We have toned down our claims for superiority of our toolbox over past or alternative solutions and focus on a simpler objective description of the provided capabilities. We nevertheless maintain that the effort we have taken thus far has already provided ample evidence for the *de facto* applicability of GravyTrain toolbox in terms of the plethora of constructed plasmids and yeast strains and the experimental data derived thereof in our recent publication on the key mechanism of autophagosomal membrane expansion in Developmental Cell (PMID: 38447569).

Several figures (e.g., Figure 3) are too dense and require simplification to enhance reader comprehension.

We have now relocated most schematic figures to the supplementary information for the advanced interested reader who may wish to employ GravyTrain in research while keeping key concepts in the main figure. As such, we hold that the revised supplementary information figures directly match in detail with the subject matter, e.g., the schematic representation of GravyTrain constructs in the context of genomic integration in yeast in Fig S3.

While yeast-related applications are well documented, the demonstration of versatility in mammalian systems is minimal and lacks experimental data.

The applicability to mammalian systems is not a key feature of GravyTrain but rather a theoretical exemplification of the potential of the community concept to expand research beyond yeast. As the community concept itself is preliminary, pending further interest on behalf of the scientific community in GravyTrain in general, we believe it is premature to make a considerable effort in experimentation in this direction beyond the construction of the actual constructs provided. Nevertheless, to ensure the applicability of the community endeavor to experimentation, we provide here (Fig R1) unpublished preliminary results of the TECPR2 proximity study conducted using the community miniTurboID TECPR2 fusion constructs to be included in our upcoming structure-function study of the differential roles of TECPR2 domains.

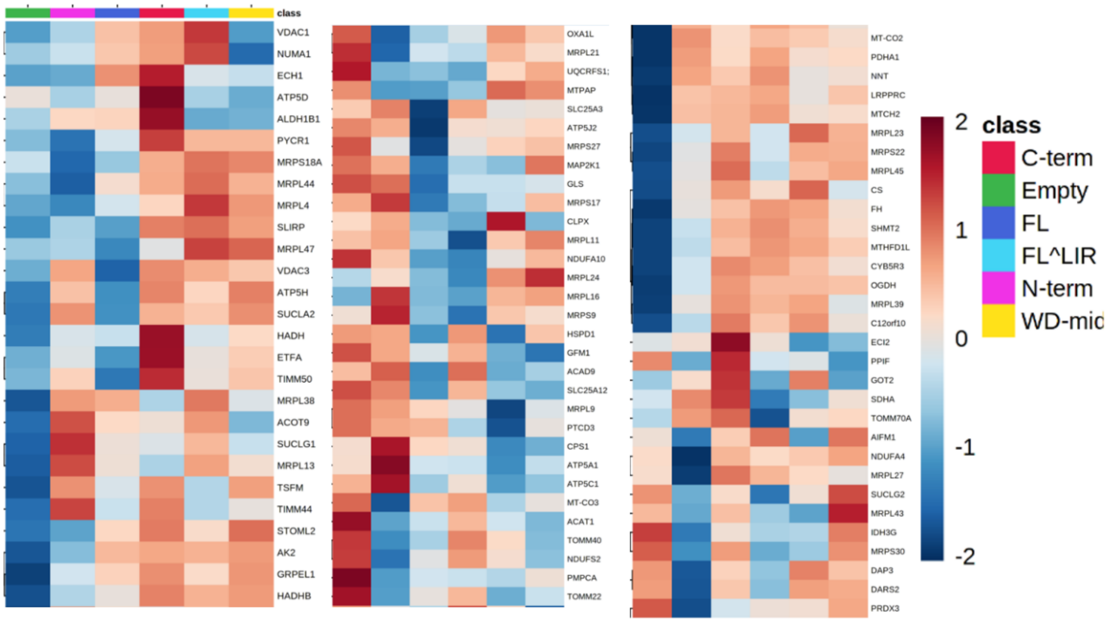

Figure R1: Proximity labelling of TECPR2 structure-function variants using miniTurboID. TECPR2 knockout MEFs were transiently transfected with indicated TECPR2 structural variant fused to miniTurboID. After pulsing with biotin, cells were lysed and Streptavidin pulldown was used to enrich labelled proteins proximal to the TECPR2 variant. Enriched proteins were subjected to LC-MS/MS at the Weizmann Institute of Science Nancy and Stephen Grand Israel National Center · for Personalized Medicine (G-INCPM). Fold-change of identified proximal proteins is shown in color-code heatmap.

The discussion overemphasized potential benefits without critically addressing limitations, scalability, or broader applications.

We have now adjusted the discussion to tone down the benefits and include potential pitfalls.

The concept of "GravyTrain Community" is intriguing but underexplored. Clearer plans for repository management and user contributions, as well as supplementary materials for reproducibility, are essential.

As stated above, further work establishing the community framework is premature, pending future interest in the toolbox. Nevertheless, we will deposit the entire toolbox plasmid collection in the publicly accessible Forchheimer Plasmid Collection of the Life Sciences Core Facility in the Weizmann Institute of Science, and key plasmids will be deposited in the international Addgene repository to allow broader accessibility to facilitate community efforts. The reproducibility of restriction free cloning is well-established prior to this publication, where we use a pair of universal primers for shuffling elements between community constructs. All DNA elements used in cloning are well documented in the supplementary information as originally submitted, whereas details pertaining to the construction of specific constructs are available upon request.

Additional response (added in response to editorial comments)

Journal Requirements:

Please review your reference list to ensure that it is complete and correct. If you have cited papers that have been retracted, please include the rationale for doing so in the manuscript text, or remove these references and replace them with relevant current references. Any changes to the reference list should be mentioned in the rebuttal letter that accompanies your revised manuscript. If you need to cite a retracted article, indicate the article's retracted status in the References list and also include a citation and full reference for the retraction notice.

We have now reviewed the list of references to ensure it is complete, correct and does not include any retracted papers.

While revising your submission, please upload your figure files to the Preflight Analysis and Conversion Engine (PACE) digital diagnostic tool, https://pacev2.apexcovantage.com/. PACE helps ensure that figures meet PLOS requirements. To use PACE, you must first register as a user.

Registration is free. Then, login and navigate to the UPLOAD tab, where you will find detailed instructions on how to use the tool. If you encounter any issues or have any questions when using PACE, please email PLOS at figures@plos.org. Please note that Supporting Information files do not need this step.

We have uploaded our figure files to the PACE tool to ensure they meet PLOS requirements.

Reviewers' comments:

Reviewer's Responses to Questions

Comments to the Author

6. Review Comments to the Author

Reviewer #1: Although the work is thorough, there are instances where the methodology and results are arranged in a way that seems repetitious or overlaps. The experimental demonstration (autophagy strain library) and the explanation of the build designs should be more clearly separated for easier reading, perhaps with the use of subheadings or summary tables inside the body of the article.

As requested by the reviewer, we have now added subheadings to the section on studying autophagy in yeast using GravyTrain for easier reading.

The manuscript heavily relies on supplemental tables and figures to explain essential concepts. Integrating some of these key schematics or summaries into the main figures would make the manuscript more self-contained and reader-friendly.

We have now integrated some of the key supplementary information into the main figure.
